# Editorial: Community series in pathogenetic mechanism and therapeutic target for inflammation in autoimmune disease, volume II

**DOI:** 10.3389/fimmu.2025.1645760

**Published:** 2025-07-18

**Authors:** Jia Li, Minrui Liang, Hai-Feng Pan, Jian Gao, Yuekang Xu, Liangjing Lu

**Affiliations:** ^1^ Department of Rheumatology, Ren Ji Hospital, Shanghai Jiao Tong University School of Medicine, Shanghai, China; ^2^ Department of Rheumatology, Huashan Hospital, Fudan University, Shanghai, China; ^3^ Institute of Rheumatology, Immunology and Allergy, Fudan University, Shanghai, China; ^4^ Huashan Rare Disease Center, Huashan Hospital, Fudan University, Shanghai, China; ^5^ Department of Epidemiology and Biostatistics, School of Public Health, Anhui Medical University, Hefei, Anhui, China; ^6^ Inflammation and Immune Mediated Diseases Laboratory of Anhui Province, Hefei, Anhui, China; ^7^ Shanghai Children’s Medical Center, School of Medicine, Shanghai Jiao Tong University, Shanghai, China; ^8^ College of Life Science and Technology, Xinjiang University, Urumqi, China

**Keywords:** pathogenetic mechanism, therapy, autoimmune disease, inflammation, target

Recent advances in basic research and clinical trials have indicated novel therapeutic approaches for autoimmune diseases. Breakthroughs in cytokine-targeting antibodies, small-molecule inhibitors, and cellular therapies are revolutionizing the management of inflammatory and autoimmune disorders (1–6). The landmark success of mRNA vaccine technology further underscores the transformative potential of nucleic acid therapeutics (7, 8). This therapeutic evolution marks a paradigm shift from broad immunosuppression to precision strategies that restore immune homeostasis, potentially enabling sustained disease remission or even cure (9).

This second volume of *Frontiers in Immunology*’s Research Topic presents nine papers that dissect molecular pathways, identify diagnostic biomarkers, and explore therapeutic strategies for autoimmune diseases. These works collectively advance our understanding of inflammation’s multifaceted roles and highlight translational opportunities for precision medicine.

Omics technologies are helpful to decipher immune dysregulation. Single-cell RNA sequencing (scRNA-seq) has emerged as a transformative tool for unraveling cellular heterogeneity. Zhao and Fang leveraged scRNA-seq to profile peripheral blood mononuclear cells (PBMCs) in type 2 diabetes mellitus (T2DM), revealing dysregulated TNF-α/NF-κB signaling and interferon-γ responses in patients. *TNFRSF1A* was identified as a core gene in T cells network. This study underscores the interplay between metabolic dysfunction and immune activation. Similarly, Yao et al. identified six hub genes (*CTSS*, *CYBB*, *FPR2*, *MNDA*, *TLR1*, *TLR8*) in pulmonary sarcoidosis through transcriptomic analysis of mediastinal lymph nodes and blood. Long non-coding RNAs (lncRNAs) are increasingly recognized as epigenetic regulators in autoimmunity. Yang et al. identified five dysregulated lncRNAs (*LINC00494*, *TSPOAP1-AS1*, *MCM3AP-AS1*, *LINC01588*, *OIP5-AS1*) in RA PBMCs, with combined diagnostic accuracy reaching an AUC of 0.920. These studies suggested that both hub genes and lncRNAs have the potential to improving the diagnostic process and understanding the mechanisms.

Wang and Liu investigated the therapeutic mechanisms of Xinfeng Capsules (XFC), a traditional Chinese medicine formulation, using clinical datasets and animal models. Interestingly, their results demonstrated that XFC modulates lncRNA DSCR9 expression to inhibit the RPLP2/PI3K/AKT signaling axis, thereby alleviating inflammatory responses and hypercoagulable states in RA. These findings elucidate a novel molecular pathway for XFC’s efficacy and provide translational insights for RA treatment.

Chronic inflammation lies at the epicenter of autoimmune pathogenesis, driving tissue destruction and systemic complications across diverse conditions. dos Santos Oliveira et al. reviewed the dual role of the Sterile Alpha and TIR Motif-Containing Protein 1(SARM1), a multifaceted component, in neuroinflammation and degeneration, proposing its enzymatic regulation as a strategy to counter neurodegeneration and inflammations.

Zhou et al. showed that granulomatous lobular mastitis (GLM) and plasma cell mastitis (PCM) pathophysiology involves multiple immune factors, such as immune cells, cytokines, prolactin, milk stasis - related hypersensitivity, lipid disorders, and inflammatory pathways. Innate and adaptive immunity have distinct functions in GLM and PCM development, during which mammary ductal epithelial cell damage occurs, intensifying breast tissue inflammation. In Seizer et al.’s case report, the authors conducted a study to track changes in endocrine and immune profiles in a rheumatoid arthritis (RA) patient during a disease flare period.

Characterized by vascular injury, inflammatory responses, fibrotic tissue accumulation, and calcinosis, systemic sclerosis (SSc) represents a heterogeneous autoimmune disease. Geroldinger-Simic et al. investigated calciprotein particles(CPP) in serum samples from 78 SSc patients and 44 controls without SSc. The authors found that reduced T50 (calciprotein crystallization test or serum calcification propensity) and increased hydrodynamic radius of secondary CPPs (CPP2) in patients with SSc, indicating a disturbed mineral buffering system in SSc.

Understanding immune mechanisms is driving the development of more effective therapeutic strategies in RA. Han et al. synthesized RA pathogenesis and emerging therapies through a cellular lens, delineating how synovial fibroblasts, osteoclasts, and immune subsets exploit NF-κB, PI3K-AKT, and MAPK pathways to perpetuate inflammation and bone destruction. Their review catalogs emerging therapies—nanoparticle delivery, immune cell therapies, traditional Chinese medicine and nucleic Acid Therapeutics—while cautioning against challenges in scalability and immune compatibility.

This review highlights the complexity of inflammatory networks in autoimmunity and outlines translational research directions. Advances from single-cell omics to lncRNA diagnostics and multimodal therapies might enhance our ability to manage autoimmune diseases. Future priorities include longitudinal biomarker validation, patient-centered trials, and interdisciplinary collaboration to translate mechanistic insights into clinical applications. Unraveling immune-tissue interactions brings precision medicine for autoimmunity closer to reality.
